# Distributive and Refractory Shock in Myxoedema Coma

**DOI:** 10.7759/cureus.75366

**Published:** 2024-12-09

**Authors:** Cristiane P Macedo, Marina Costa, Isabel Eira, Rui Castro, Pedro Silveira

**Affiliations:** 1 Internal Medicine, Unidade Local de Saúde de Coimbra, Coimbra, PRT; 2 Intensive Care Unit, Hospital de Braga, Braga, PRT

**Keywords:** distributive shock, hypothyroidism, medical emergency, myxoedema coma, refractory shock

## Abstract

Myxoedema coma is a rare medical emergency, presenting even less commonly without sepsis and with the diagnosis of distributive shock. Reports of catecholamine-refractory shock are scarce.

This report describes the case of a 54-year-old male, who presented to the emergency department with altered mental status. He was found to be hypothermic, hypotensive, and bradycardic, with poor peripheral perfusion and oedema of the face and hands. On initial evaluation, the patient was found to have acute renal failure, hyponatraemia, hyperkalaemia, and findings suggestive of metabolic acidosis. Clinical examination confirmed a suspected diagnosis of distributive shock with multiorgan dysfunction, refractory to volume, and vasopressor support. The patient was admitted to the intensive care unit for treatment and organ support. Further diagnostic studies revealed an elevated thyroid-stimulating hormone level and decreased T3 and T4, favouring the diagnosis of myxoedema coma. Hydrocortisone and levothyroxine were initiated intravenously for the treatment of suspected myxoedema coma, resulting in rapid clinical improvement.

## Introduction

Myxoedema coma is a medical emergency and a rare condition with an incidence of 0.22 cases per million per year in Spain with similar data worldwide [1,2]. It has a high mortality rate (25-60%) without early diagnosis and proper treatment [3]. 

Myxoedema coma is characterised primarily by reduced mental clarity and hypothermia. Additional common findings include hypotension, bradycardia, hyponatraemia, hypoglycaemia, and hypoventilation. Patients may exhibit puffiness in the hands and face, along with features such as a thickened nose, swollen lips, and an enlarged tongue, which arise from non-pitting oedema caused by abnormal accumulation of albumin and mucin within the skin and other tissues. Clinical signs related to the underlying acute condition triggering myxoedema coma, such as infection or myocardial infarction, may also be evident [4]. Precipitating factors may also include severe hypothyroidism and exposure to cold temperatures, with female patients being more commonly affected [2,5]. Importantly, in cases of infection, a febrile response might be absent due to impaired thermogenesis mediated by thyroid hormones [4].

## Case presentation

A 54-year-old male presented to the emergency department (ED) during a hot summer day with altered mental status, confusion, and obnubilation. The patient was brought from home, via emergency medical transport due to reported confusion and abdominal pain. Unfortunately, a more comprehensive history was unobtainable with no reported medications or prior medical diagnosis available. On emergency room examination, a primary survey was made. His airway was unobstructed, he was tachypnoeic with a respiratory rate of 26 cycles per minute, with no other signs of respiratory distress, and his oxygen saturation was 96% on room air. His peripheral pulses were not palpable and blood pressure (BP) was not measurable. He presented mottling grade 3, and rapid resuscitation with crystalloid fluids was initiated. His heart rate was 70 beats per minute and his tympanic temperature was 34.5ºC consistent with hypothermia. An immediate arterial blood gas (ABG) was performed, with findings suggestive of metabolic acidosis, hyponatraemia, and hyperkalaemia without elevated lactic acid. Repeated BP after a few minutes of fluids was 50/30 mmHg. A point-of-care ultrasound was performed in the ED and showed preserved systolic biventricular function and no cardiac tamponade or wall motion abnormalities. 

Due to his history of abdominal pain, we initially suspected an abdominal aortic rupture as the aetiology of this shock. The thoracoabdominal-pelvic computerised tomography scan did not show any rupture or mechanical justification for the shock, thromboembolism, or infection site.

During observation in the ED, he became bradycardic with an electrocardiogram confirming bradycardia of 47 bpm with a wide QRS complex. Urine output remained absent and repeated ABG revealed hyperlactataemia of 3.2 mmol/L and worsening of metabolic acidaemia with a bicarbonate of 10 mmol/L. After initial vasopressor support with adrenaline and ephedrine bolus, the patient was started on norepinephrine to a maximum dose of 1.3 mcg/kg/min. His blood tests showed acute renal failure, hyperkalaemia, hypocalcaemia, hyponatraemia, elevated C-reactive protein, and leucocytosis (Table 1).

**Table 1:** Analytical study at admission pCO_2_: partial pressure of carbon dioxide; pO_2_: partial pressure of oxygen.

Parameter	Result	Reference range
Haemoglobin (g/dL)	15.0	13.5-17.0
Leukocytes (x10^3^/uL)	22.4	4.0-11.0
Platelets (x10^3^/uL)	395	150-400
Urea (mg/dL)	300	19-49
Serum creatinine (mg/dL)	13	0.70-1.20
Potassium (mmol/L)	7	3.5-5.1
Sodium (mmol/L)	128	136-145
Calcium (mmol/L)	0.98	1.15-1.35
Phosphorus (mg/dL)	12.6	2.4-5.1
Magnesium (mg/dL)	24	16-26
C-reactive protein (mg/L)	146.1	<5.0
pH	7.37	7.37-7.45
pCO_2 _(mmHg)	21.7	35.0-46.0
pO_2_ (mmHg)	75.8	70.0-100.0
Bicarbonate (mmol/L)	12.5	21-26
Lactate (mmol/L))	1.63	0.5-2.0

**Figure 1:**
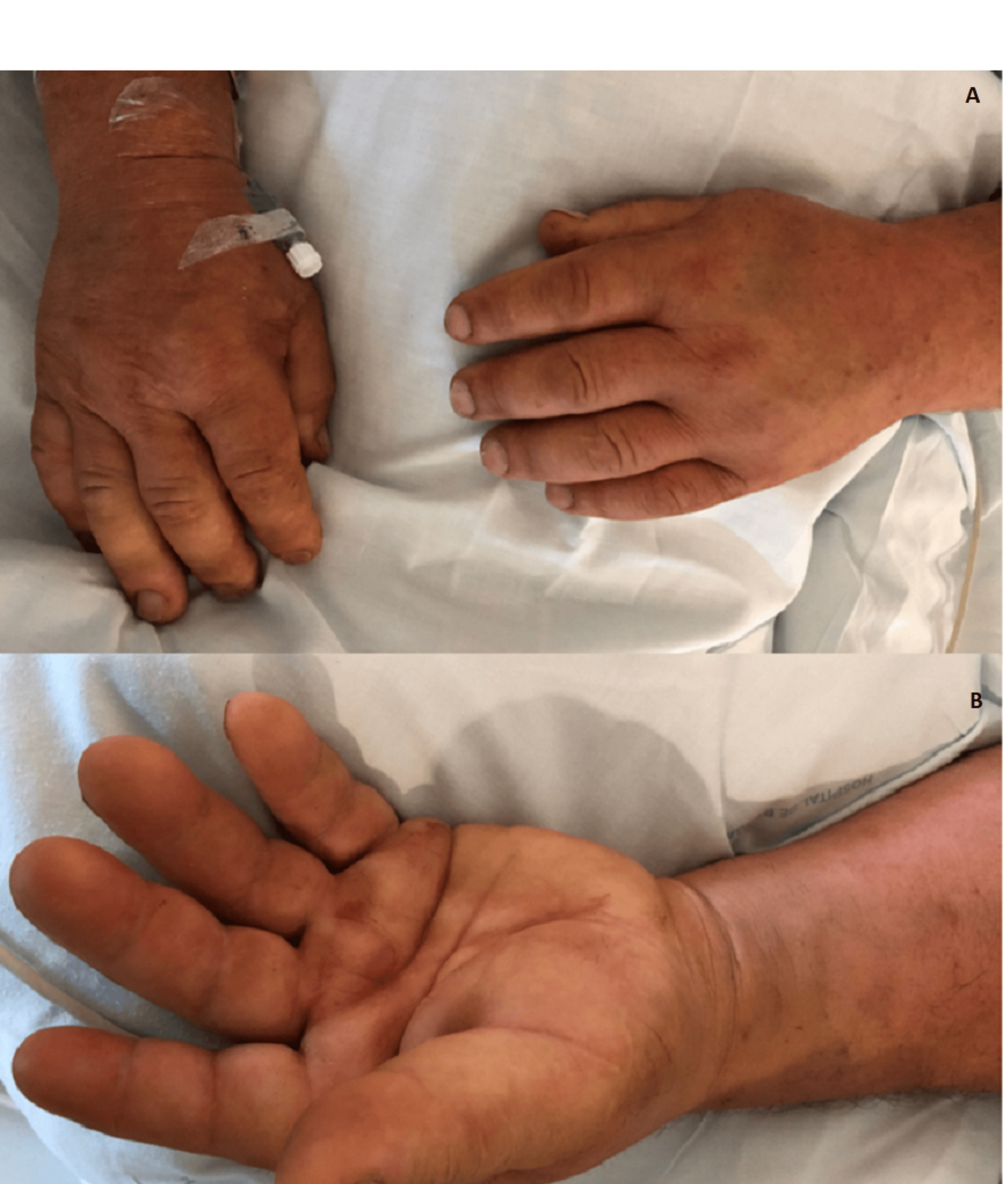
Non-pitting oedema Non-pitting oedema in a patient with hypothyroidism. (A) Dorsal view of both hands showing swelling and thickening, consistent with non-pitting oedema. (B) Palmar view further demonstrates the characteristic features of non-pitting oedema, including diffuse swelling without indentation upon pressure. These findings are hallmark signs of severe hypothyroidism.

At this point, we became aware he had a history of hypertension and dyslipidaemia, although no additional medical records were available including medication. We had excluded cardiogenic, obstructive, and hypovolemic shock, and so we had a patient with a distributive shock with neurologic, cardiovascular, and renal dysfunctions. Thinking of a possible contribution of adrenal insufficiency, a bolus of hydrocortisone 200 mg was administered, resulting in an improvement of haemodynamic dysfunction. After stabilisation, we noticed that he had non-pitting oedema in his face and hands (Figure 1).

He was admitted to the intensive care unit (ICU) with the diagnosis of distributive shock of undetermined origin. We started continuous venovenous hemodiafiltration for suspicion of uremic encephalopathy. Further blood tests revealed high levels of thyroid-stimulating hormone (TSH) and low levels of free T4 and T3 (Table 2). Cortisol was elevated and adrenocorticotropic hormone (ACTH) was normal excluding concomitant adrenal insufficiency, leaving the diagnosis of primary hypothyroidism presenting as myxoedema coma. Levothyroxine was administered intravenously (IV) in the loading dose of 200 mcg. After starting hormone replacement therapy, the patient showed rapid clinical improvement, with recovery of his normal mental status and resolution of cardiovascular dysfunction in just a few hours, and renal dysfunction overnight with the return of spontaneous urine output.

**Table 2:** Further analytical study ACTH: adrenocorticotropic hormone; TSH: thyroid-stimulating hormone; T4: thyroxine; T3: triiodothyronine.

Parameter	Result	Reference range
TSH (uUI/mL)	177	0.55-4.78
Free T4 (ng/dL)	0.39	0.89-1.76
T3 (pg/mL)	1.10	2.3-4.2
Cortisol (ug/dL)	175.36	4.3-22.4
ACTH (pg/mL)	11.30	<46

After recovery, the patient admitted having discontinued his medication with levothyroxine one month before coming to Portugal on holidays. Levothyroxine was titrated to a daily dose of 150 mcg in the next few days. Thinking about other possible precipitating events as infection, he had a clinical history of diarrhoea without bloody stools and elevated inflammatory markers, reason why he was treated empirically with ceftriaxone for five days. However, his inflammatory markers returned to normal levels in 48 hours and his blood and urine microbiological cultures came out negative.

He was discharged from the ICU after three days. Levothyroxine was titrated to 150 mcg IV per day. The patient was discharged home and referred to endocrinology outpatient consultation.

## Discussion

Hypothyroidism has a range of clinical presentations and is usually detected before achieving this severity. Typically, a precipitating event disrupts the homeostasis maintained in hypothyroid patients through various neurovascular adaptations [6]. In this case, we had several clinical clues that could lead us to the correct diagnosis. The patient had bradycardia, which is one of the key features of severe hypothyroidism, hypothermia, hyponatraemia, and face and hands oedema [7]. In suspicion of this diagnosis, thyroid hormone should be given as well as hydrocortisone in stress doses once adrenal insufficiency is usually associated [8-10].

In fact, high doses of thyroid hormone replacement appear to increase the risk of precipitating fatal tachycardia or myocardial infarction, with a safe dose being less than 500 mcg per day. It is also known that pituitary-adrenal function is impaired in severe hypothyroidism and the restoration of a normal metabolic rate with exogenous thyroid hormones may precipitate adrenal insufficiency. The exact dose of replacement is not established; however, some authors indicate a large initial dose of 300-500 mcg of T4 and if the patient does not show clinical improvement in 24 hours, T3 should be added in an initial dose of 10-25 mcg followed by 2.5-10 mcg every 8 hours [6]. 

The pathogenesis of myxoedema coma involves several organ systems. When serum T4 decreases, it results in a lower intracellular level of T3 which directly affects the central nervous system, causing altered mental status. This also results in decreased thermogenesis and consequently hypothermia. Cardiac inotropism and chronotropism, as well as sensitivity to adrenergic stimuli, are decreased, leading to reduced cardiac output and consequent generalised vasoconstriction, which may result in a drop in blood pressure and culminate in cardiogenic shock [11]. 

Few reports describe refractory shock due to myxoedema coma, and these usually refer to cardiogenic shock [2-5,11]. In our case, our patient maintained normal cardiac output and the absence of pleural effusion. 

There are some cases described of catecholamine refractory shock, particularly in children [2,12]. In this case, myxoedema presented with a distributive shock without sepsis which is extremely rare. 

## Conclusions

This report highlights the necessity for heightened clinical awareness of such atypical presentations to improve outcomes in this rare, life-threatening condition. Prompt initiation of treatment is essential to reduce mortality in these patients.

The findings contribute valuable insights to the limited literature on myxoedema coma and its management in the context of distributive shock, supporting the need for further studies to optimise diagnostic strategies and treatment protocols.
